# Current treatment of chronic hepatitis B: Clinical aspects and future directions

**DOI:** 10.3389/fmicb.2022.975584

**Published:** 2022-09-08

**Authors:** Minmin Zhu, Hui Wang, Tao Lou, Pian Xiong, Jiebing Zhang, Lele Li, Yuchao Sun, Yingping Wu

**Affiliations:** ^1^The Fourth Affiliated Hospital of Zhejiang University School of Medicine, Jinhua, China; ^2^International Institutes of Medicine, Zhejiang University, Jinhua, China

**Keywords:** HBV infection, ALT, treatment, immune tolerant, withdrawal, retreatment

## Abstract

Hepatitis B virus (HBV) infection is a public health threat worldwide, and there is no direct treatment yet available. In the event of infection, patients may present liver cirrhosis and cancer, which threaten the patients’ health globally, especially in the Asia-Pacific region and China. In 2019, Chinese hepatopathologists updated the 2015 Guidelines for the Prevention and Treatment of Chronic Hepatitis B as the clinical reference. The other versions formulated by the American Association for the Study of Liver Diseases (2018 AASLD guidelines) (AASLD, 2018), European Association for the Study of the Liver (2017 EASL guidelines) (EASL, 2017), and Asian-Pacific Association for the Study of the Liver (2015 APASL guidelines) (APASL, 2015) also provide clinical guidance. However, there are still some issues that need to be addressed. In the present study, the following aspects will be introduced successively: (1) Who should be treated in the general population according to the guidelines; (2) Treatment of specific populations infected with HBV; (3) Controversial issues in clinical practice; (4) Perspective.

## Introduction

According to the World Health Organization (WHO) statistics, approximately 0.257 billion people are infected with the Hepatitis B virus (HBV) worldwide, with around 0.88 million deaths annually (Trépo et al., 2014; Valery et al., 2018), which causes a huge medical and economic burden. Among 70 million HBV-infected patients in China, 20–30 million people had CHB (chronic hepatitis B) (Liu et al., 2019). Viral hepatitis is expected to be eliminated by 2030. Therefore, significant efforts have been made worldwide to provide practical and standardized guidelines for preventing, diagnosing, and treating HBV infection. However, not all patients requiring treatment are within the guidelines of treatment criteria; thus, it is essential for all the candidates from the HBV-infected population.

### Candidates in the general population

Antiviral treatment is an effective therapeutic strategy for CHB patients that efficiently suppresses HBV replication, decreases inflammatory necrosis in the liver, reduces the incidence of liver cirrhosis and related complications, and reduces the fatality rate associated with hepatocellular carcinoma (HCC) and other liver diseases. In the 2019 China guidelines (Chinese Society of Infectious Diseases, 2019), HBV infection is divided into four phases: immune tolerance, immune clearance, immune control, and immune reactivity, and it is different from the 2015 version (Hou and Lai, 2015; Table 1). Additionally, the 2019 China guidelines eased the restrictions on indications for antiviral therapy, and reducing the demand for HBV-DNA load. Conversely, the HBV-DNA load is considered for the performance of antiviral therapy in the 2018 guidelines (Terrault et al., 2018) updated by the 2018 AASLD guideline and the 2017 EASL guidelines (Table 2). For the treatment of HBV infection with normal ALT (alanine aminotransferase), antiviral therapy is recommended in patients > 30-years-old with a family history of liver cirrhosis or cancer in the 2019 China guidelines. In another case >30-years-old without a family history of liver cirrhosis or cancer, a hepatic biopsy was recommended. Although we can refer to many guidelines, there are many patients failed to fulfill the criteria for treatment at follow-up and eventually developed liver fibrosis, cirrhosis, and cancer (Wang et al., 2013; Alam et al., 2014).

**TABLE 1:** Comparison between 2015 (China) and 2019 (China) guidelines.

Guidelines	ALT level or Liver inflammation/fibrosis degree	Other	Timing of assessment for liver inflammation and fibrosis (liver biopsy or non-invasive examination)
2015	Continuous ALT > 2 ULN, otherwise, ≥Grade 2 liver inflammation or fibrosis	Liver cirrhosis (regardless of ALT/HBeAg), aggressive anti-viral treatment	Normal ALT, age > 30 years, a family history of liver cirrhosis or liver cancer; ALT = 1–2 ULN, age > 30 years
2019	Continuous ALT > 1 ULN, excluding other causes; Normal ALT, ≥Grade 2 liver inflammation and (or) fibrosis	Liver cirrhosis decompensation, HBV DNA+, anti-viral treatment; liver cirrhosis decompensation, HBsAg+, anti-viral treatment; age > 30 years, a family history of liver cirrhosis or HBV-related liver cancer, anti-viral treatment; HBV-related extrahepatic manifestation	Normal ALT, age > 30 years

**TABLE 2:** Indications for chronic hepatitis B (CHB) treatment in 2017 (EASL) and 2018 (AASLD) guidelines.

Guidelines	HBeAg+		HBeAg-	
	HBV DNA level	ALT	HBV DNA level	ALT
2017 (EASL) (ULN 40 U/L)	≥2,000	>ULN and (or) at least moderate liver necroinflammation or fibrosis	≥2,000	>ULN and (or) at least moderate liver necroinflammation or fibrosis
	≥20,000	>2 ULN, regardless of the degree of fibrosis	≥20,000	>2 ULN, regardless of the degree of fibrosis
2018 (AASLD) (Male, ULN 35 U/L; Female, ULN 25 U/L)	≥20,000	>2 ULN, or significant liver histologic transformation	≥2,000	>2 ULN, or significant liver histologic transformation

### Patients with a normal level of alanine aminotransferase

Hepatitis B virus infection is a dynamic process characterized by fluctuations in alanine ALT, which might hint toward immune-mediated virus clearance (Ghany et al., 2020). Since the ALT level is not always indicative of inflammation in the liver, patients with normal ALT levels can present inflammation and fibrosis on liver biopsy. Thus, ALT is used as a substitute for liver inflammation when liver histology is a failure (Wu et al., 2019, 2020; Fang et al., 2022). But the challenge in defining the ULN (upper limits of normal) of ALT is the difficulty of including totally healthy subjects without liver diseases, especially MAFLD (Metabolic-Associated Fatty Liver Disease), the leading cause of liver disease worldwide (Tampi et al., 2020). An Italy study reveals that Male sex, body mass index, glucose, lipids, ferritin, hypertension, and younger age are independent predictors of ALT (Valenti et al., 2021). Many hepatologists call for the adjustment of the ULN of ALT (Park et al., 2008; Chen et al., 2010). In 2019 China guidelines, the ULN remains constant at 50 U/L in males and 40 U/L in females; however, many studies have recommended rational values as 35 U/L in males and 23 U/L in females (Harbour and Miller, 2001; Zheng et al., 2012; Shang et al., 2013). In 2018 AASLD guidelines, the ALT ULN is modified as 35 U/L in males and 25 U/L in females, as described previously (Lee et al., 2010; Ruhl and Everhart, 2012; Terrault et al., 2018). In 2017 EASL guidelines and 2015 APASL guidelines, the ALT ULN is 40 U/L in both males and females (Ruhl and Everhart, 2012; Sarin et al., 2016; European Association for the Study of the Liver, 2017). Therefore, whether patients have normal ALT levels partially depends on the ULN. The ULN values mentioned in this study are consistent with those in the literature.

### Hepatitis B virus-infected patients with normal alanine aminotransferase

Patients infected with HBV but have normal ALT levels may exhibit manifestations of liver fibrosis and inflammation upon histological examination (Chang et al., 2021; Liu et al., 2022). Previous studies have shown that people with normal ALT levels develop moderate liver tissue inflammation (16.8–40%) and moderate liver fibrosis (24.2–35.9%) (Ormeci et al., 2016; Tan et al., 2017; Choi et al., 2019; Liu et al., 2022), which are associated with a high risk of progression to liver cirrhosis and cancer. In a retrospective study consisting of 327 HBV DNA + CHB patients who underwent liver biopsy, significant differences were detected between high-normal ALT (20 U/L < ALT < ULN) and low-normal ALT (ALT ≤ 20 U/L) groups in liver inflammation and fibrosis (*P* < 0.01). The rate of significant liver tissue inflammation is 44.6 and 26.5% corresponding to 20 U/L < ALT < ULN and ALT ≤ 20 U/L, respectively (Duan et al., 2021). Another study (Choi et al., 2019) demonstrated that patients with normal ALT have a significantly decreased risk of long-term liver cancer and required liver transplantation after antiviral treatment compared to those without treatment. Therefore, in cases with normal ALT levels, a comprehensive examination is essential, especially in the condition of liver fibrosis and inflammation, to provide antiviral treatment in a time-dependent manner.

### Immune tolerant chronic hepatitis B

Immune tolerance is a status presenting HBeAg+, high viral replication, and normal ALT. It was put forward as a lack of specific immune response to HBV due to the immature immune system during infancy. No treatment is recommended for such cases in the current guidelines, as it is speculated that patients in the immune tolerance phase have a low risk of progression to liver cirrhosis or cancer (Cornberg et al., 2019; Lee et al., 2020). Moreover, ineffective treatment may lead to a low e-antigen seroconversion rate (Wu et al., 2010; Jeng and Lok, 2021). Patients with immune tolerance are mostly teenagers and readily develop drug resistance because of poor compliance after long-term antiviral treatment (Dolman et al., 2018).

Definitions of IT-CHB differ among guidelines. In 2018 AASLD guidelines, IT-CHB is defined by HBV DNA > 10^6^ IU/mL and normal ALT, while in 2017 and 2019 China guidelines, IT-CHB is defined by HBV DNA > 10^7^ IU/mL and normal ALT. However, the ULN of ALT remains controversial with limited value in distinguishing patients experiencing immune tolerance, as the patients are still at risk of liver inflammation or fibrosis when their ALT levels are defined as normal under the lowest ULN standard (Ormeci et al., 2016; Tan et al., 2017; Choi et al., 2019; Chang et al., 2021; Duan et al., 2021; Liu et al., 2022). Hong et al. (2015) speculated that innate immune cell maturation and helper T lymphocyte development existed before intrauterine exposure to HBV during infancy, while another study discovered HBV-specific T lymphocytes in that period (Bertoletti and Ferrari, 2016). Presumably, the immune tolerance phase refers to a status of mild immune response that is difficult to be recognized, and thus, an accurate diagnosis was challenging. A previous study also found the presence of HBV-DNA integration into the host chromosome and the clonal expansion of stem cells in cases of IT-CHB (Mason et al., 2016). A high level of HBV replication is regarded as an independent risk factor for disease progression to cirrhosis and HCC. Additionally, some experts speculated that liver cirrhosis or cancer progresses dynamically and continuously (Kennedy et al., 2017; Sun et al., 2018). Given the fact that the age of CHB onset is mainly after 30 years, the disease progresses for many years before the onset. Furthermore, there is a decreased transmission in immunotolerant patients bearing a high viral load after antiviral treatment. In all guidelines, no treatment is recommended for immunotolerant patients; instead, antiviral therapy is clarified as alleviating liver tissue inflammation and fibrosis and delaying and decreasing the risk of cirrhosis, relevant complications, and cancer. In patients with IT-CHB, aggressive antiviral treatment is beneficial in lowering the risk of liver cirrhosis and cancer. Whether IT-CHB patient needs treatment requires both individualized and comprehensive assessment implicating multiple factors, such as patient age, willingness to receive treatment, risk of disease progression, virus genotype (Yang et al., 2008; Wong et al., 2013), family history, and lifestyle.

### Treatment of specific populations infected with hepatitis B virus

As mentioned above, a family history of HBV-related cirrhosis or HCC is a major factor for treatment. Typically, when people develop cirrhosis or HCC, treatment should be positive, which has been described elsewhere. The following groups are highlighted in this review.

### Coinfection

**FIGURE 1:**
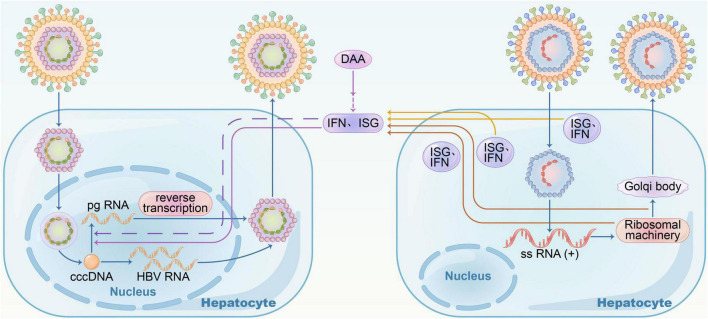
Hepatitis B virus– hepatitis C virus (HBV–HCV) coinfection. In HBV-HCV coinfection, HBV replication was suppressed by interferon response resulting from HCV replication; when treated with DAA, HBV RNA increased, and there was no direct virus-virus interference.

Hepatitis B virus, human immunodeficiency virus (HIV), and hepatitis C virus (HCV) share the same modes of transmission, and hence, superinfection is rather common. HBV coinfected with HCV is likely to progress to cirrhosis, chronic and fulminant hepatitis, and HCC in the liver (Squadrito et al., 2013; Pol et al., 2017). All HBsAg-positive patients should be screened for anti-HCV, especially those at a high risk of infection, such as drug injections and male homosexuality. In coexisting HBV and HCV cases, HBV replication differs from HBV mono-infection and is prevented by IFN (interferon) response from HCV replication compared to direct interactions between viruses (Cheng et al., 2020; Shih and Liu, 2020). In HBV-HCV superinfection, the presence of HBV infection does not affect HCV replication. Cheng et al. reported that ISG (Interferon-stimulated gene) expression mediated by HCV infection induced HBV suppression; on the contrary, both HBV and HCV viruses showed high levels of replication when ISG expression was blocked. Specifically, CXCL10 may be a marker of significant IFN response in HBV-HCV coinfection and HCV clearance. In clinical practice, CHB patients who are RNA-positive for HCV should be treated with DAA (direct-acting antiviral) plus anti-HBV therapy, according to almost guidelines (Figure 1). Moreover, monitoring serum HBsAg and HBV DNA levels monthly during the process in patients who receive DAA with HBsAg-negative and hepatitis virus core antigen-positive (anti-HBc+) is essential in case of HBV reactivation.

In HIV-HBV coinfection cases, early initiation of ARVT is recommended, irrespective of the CD4 count. According to the 2019 China guidelines, the therapy included two types of anti-HBV drugs to avoid HIV resistance to NAs (Chinese Society of Infectious Diseases, 2019).

### Patients who receive immunosuppressive or cytotoxic therapy

While receiving immunosuppressive or cytotoxic therapy, patients with HBsAg-positive, anti-HBc-positive or HBsAg-negative, and anti-HBc-positive have the possibility of HBV reactivation. Thus, it is necessary to screen for HBsAg before therapy. In the case of HBsAg-positive, patients should receive anti-HBV prophylaxis before immunosuppression or cytotoxic therapy or consecutively. If HBsAg-negative, HBcAb+ patients undergoing anti-CD20 antibody therapy (for example, rituximab) or stem cell transplantation, should accept anti-HBV prophylaxis for at least 18 months (12 months suggested in 2018 AASLD) after immunosuppressive therapy, as the rate of HBV reactivation is high, according to 2019 Chinese guidelines.

### Pregnancy-related situations

Screening for HBsAg in childbearing age women planning a pregnancy and pregnant women is strongly recommended. When they fulfill the treatment criteria, antiviral prophylaxis with tenofovir disoproxil fumarate (TDF) is a preferred agent. NAs used for the prevention of HBV perinatal transmission in a pregnant woman at weeks 24–28 of gestation with normal ALT but positive HBeAg and high levels of viremia (HBVDNA > 2 × 10^5^ IU/mL) can be stopped immediately or within 3 months after delivery under timely monitoring (Chinese Society of Infectious Diseases, 2019; Kumar et al., 2022).

### Organ transplantation

Solid organ transplant recipients are susceptible to HBV. Donor and recipient HBV infection status and perioperative management are the main determinants of susceptibility. Antiviral therapy and HBIG might reduce reactivation or reinfection after transplantation (Chinese Society of Organ Transplantation and Chinese Medical Association, 2019).

### Treatment of chronic hepatitis B in children

Vaccination against HBV among young Chinese individuals decreased the HBV infection rate. In recent years, several studies reported gradually increasing the horizontal transmission of HBV in early childhood, and family members may be the main source of infection. Interferons and NAs are the potential treatment options according to the age of young people (Pan et al., 2020).

### Controversial issues in clinical practice

#### Nucleo(t)ide analogs withdrawal and retreatment

Oral nucleo(t)ide analogs (NAs) used in first-line treatment, such as TDF, entecavir (ETV), and tenofovir alafenamide fumarate (TAF), have strong anti-virus effects, fewer side effects, convenience, and low resistance. However, long-term duration, especially >10 years of administration, requires intensive focus on drug safety and may result in reduced compliance in patients (Ford et al., 2018; Shin et al., 2018). Drug withdrawal can be considered in HBeAg+ patients after e-antigen seroconversion and consolidation therapy, as recommended by guidelines (2019 China, 2018 AASLD, 2017 EASL). Conversely, for HBeAg- patients, the 2019 China guidelines recommended drug withdrawal upon serum HBsAg disappearance and HBV DNA below the limit of detection, and the 2018 AASLD guidelines recommended at least 2 years of viral inhibition and consolidation therapy, while the 2017 EASL guidelines recommended a minimal 3-year viral inhibition. The 2015 APASL guidelines recommended drug withdrawal upon serum HBsAg disappearance, followed by 12-month consolidation therapy or undetectable HBV DNA and a minimum of 2 years of treatment.

Whether antiviral treatment can be discontinued in HBeAg- patients is yet controversial. Clinical practice has identified a potential risk of recurrence, decompensation of liver cirrhosis, liver failure, and death after drug withdrawal. The recurrence after drug withdrawal can be defined as a VR (virological relapse): viral rebound plus HBV DNA > 2,000 IU/mL; CR (clinical relapse): VR plus ALT > 2 × ULN; hepatitis flare: VR plus ALT > 5 × ULN. Due to the putative consequences, physicians face enormous pressure to discontinue the drugs in HBeAg- patients, while in recent years, the safety of the management has been promoted. For example, in a cohort study involving 691 CHB patients (including 308 patients with liver cirrhosis) (Jeng et al., 2018), 3-year follow-up witnessed CR in 419 (61%) patients, hepatitis flare in 280 (41%) patients, total bilirubin > 2 mg/dL in 72 (10%) patients, PT (prolonged anti-pertussis toxin), and INR (International Normalized Ratio) > 1.5 in 16 (2%) patients. The incidence of decompensated liver cirrhosis was reported as 0.28% at 155-week mean follow-up duration, including 0% in CHB patients and 2.85% in liver cirrhosis patients. In a randomized controlled trial of 67 patients (2:1 to stop or continue NA therapy) over 72 weeks, 21% of patients developed an ALT > 10 × ULN, and another 10% had ALT > 5 × ULN; most patients relapsed after discontinuation, but neither hepatic decompensation nor mortality occurred (Liem et al., 2019). Some studies indicated that drug withdrawal is feasible and safe in HBeAg- CHB patients (Berg et al., 2017; Liaw, 2019), and other studies put forth the positive clinical value of drug withdrawal for HBsAg clearance (Chen et al., 2018; Papatheodoridis et al., 2018). For instance, the small-scale, randomized controlled trial by Berg et al. (2017) observed a 19% HBsAg clearance rate during the 3-year follow-up period in CHB patients after discontinuation of TDF-based antiviral therapy, while this rate in patients persistently receiving TDF was 0%. This finding was consistent with the study by Jeng et al. (2018), wherein benefits of HBsAg clearance without retreatment were observed after drug withdrawal. In the study of Jeng et al., 42 patients were free from HBsAg seroclearance during a median off-therapy follow-up of 155 weeks. Approximately 1.78% of the annual incidence is attributed to the 6-year cumulative incidence. Clinically relapsed patients who did not receive treatment had a 7.34-fold higher incidence of HBsAg clearance than those who received treatment. Thus, it may be possible to achieve a functional cure after an untreated clinical relapse that triggers sufficient immune control. In a prospective study (Rinker et al., 2018), 15 HBeAg-negative CHB patients on long-term NA treatment underwent NA discontinuation. After ceasing HBV therapy, a relapse of active HBV replication might trigger an immunological environment that influences T cell phenotypes and increases HBV-specific T cell responsiveness *in vitro* after ceasing HBV therapy. Additionally, blocking PD-L1 (programmed cell death protein 1) may further strengthen these T cell responses to HBV.

In 2021, APASL introduced Guidance on Stopping Nucleo(t)ide Analogs in Chronic Hepatitis B Patients (Kao et al., 2021), wherein NA withdrawal is recommended in HBeAg+ patients experiencing e-antigen seroconversion and consecutive 3-year treatment and in HBeAg- patients who have had undetectable HBV DNA and received treatment for at least 3 years. Accumulating evidence proposed that a finite course of antiviral therapy is a more viable option than pursuing HBsAg disappearance in HBeAg- patients. Nevertheless, these findings need to be substantiated further. In addition to HBsAg clearance, timely monitoring and examination for HBV DNA, ALT, and PT are necessary to actively assess the risk for recurrence and disease progression. Low HBsAg level after drug withdrawal (especially <100–150 U/mL), low baseline HBV DNA load, and prolonged antiviral therapy are recognized as low-risk factors for disease recurrence. For the past few years, several efforts have been made to identify new predictive factors for recurrence. Reportedly, low HBcAg and HBV RNA levels after drug withdrawal are predictive of a low risk of disease recurrence (Carey et al., 2020; Sonneveld et al., 2022). Fan et al. (2020) demonstrated that after 4 years of NA withdrawal, HBV RNA + patients were more likely to have CR and VR than those with HBV RNA below the limit of detection at the time of withdrawal. Moreover, HBcAb (<100 U/mL), host factors, and HBV genotype were also associated with CHB recurrence (Chen et al., 2018; Rivino et al., 2018; Tseng et al., 2018; Kuo et al., 2019). Currently, there is no guidance and consensus for retreatment of recurrence after drug withdrawal. Berg et al. (2017) suggested that patients who experienced recurrence after drug withdrawal and conformed to one of the following five criteria were recommended to undergo retreatment: (1) Two consecutive total bilirubin and ALT > ULN; (2) ALT > 10 × ULN; (3) 2 × ULN < ALT ≤ 5 × ULN for minimum 12 consecutive weeks; (4) 5 × ULN < ALT ≤ 10 × ULN for minimum four consecutive weeks; (5) 2 s (s) extension of PT, which cannot be corrected by vitamin K supply, and increased ALT. In clinical practice, the risk of disease progression should be highlighted before retreatment of recurrence after drug withdrawal to decrease the incidence of decompensated liver cirrhosis, liver failure, and death. Also, defining the potential predictors and retreatment criteria in order to optimize the benefits of HBsAg loss and minimize the adverse effects of severe hepatitis flare after stopping NA therapy is imperative.

#### Low level viremia

In the 2019 China guidelines, with the exclusion of patient compliance and test errors, poor therapeutic response is defined by HBV DNA > 10^3^ IU/mL after 48-week oral first-line antiviral treatment in CHB patients without liver cirrhosis, while HBV DNA > 10^3^ IU/mL after 24-week administration of first-line antiviral agents in CHB patients with liver cirrhosis. Drug regimens were adjusted according to the previous administrations and drug resistance (Table 3). Due to the advancement in the testing methods, HBV DNA < 10^3^ IU/mL, even within 20–100 IU/mL, can be readily achieved in clinical practice. LLV first received attention in the 2018 AASLD guidelines. Some researchers recommended that patients with HBV DNA < 2 × 10^3^ IU/mL after a minimal 48-week of first-line antiviral therapy are considered to have LLV, with the exclusion of patient compliance, viral resistance mutations, and test errors (Chen et al., 2006; Kim et al., 2017). The incidence of LLV might be associated with the cccDNA (covalently closed circular DNA) of HBV (Dandri and Petersen, 2016). cccDNA has a long half-life that cannot be completely eradicated from liver cells, leading to persistent low-level HBV DNA in the serum. A Korean study reported persistent or intermittent LLV in 37.9% of CHB patients in the cohort of 996 patients receiving initial first-line antiviral treatment (Kim et al., 2017). Studies from China also reported that the incidence of LVV was >30% (Sun et al., 2020; Zhang et al., 2021). Thus, it was confirmed that persistent LLV is associated with a high risk of disease progression to liver fibrosis or cancer (Kim et al., 2017; Sun et al., 2020). Currently, no medicine-based evidence explicates the utilization of the current anti-HBV agents to minimize the incidence of LLV and related side effects. In addition, no effective solutions have yet been proposed by the guidelines. As recommended by the Expert Opinion on Expanding Anti-HBV Treatment for CHB in China, the first-line antiviral agents should be adopted in a timely manner in CHB patients receiving non-first-line agents, while in patients who received the first-line agents, other first-line agents, or combination strategies (two first-line agents or combination with peginterferon) are recommended. A previous study demonstrated that LLV patients receiving ETV alone or in combination for 48 weeks achieved complete virologic suppression after replacement by TAF (HBV DNA < 20 IU/mL) (Ogawa et al., 2020). Similarly, another study suggested that ETV-naïve LLV patients could benefit from switching to TAF (Li et al., 2021). Nonetheless, all these studies are limited in experimental methods and sample size. Thus, in the future, prospective, double-blind, randomized controlled trials are required to substantiate these findings.

**TABLE 3:** Recommendations for salvage therapy in resistant patients in 2019 (China) guideline.

Type of resistance	Recommended drugs
LAM or LdT-resistance	TDF or TAF
ADV-resistance, without a history of LAM or LdT administration	ETV, TDF, or TAF
ADV-resistance, LAM/LdT-resistance	TDF or TAF
ETV-resistance	TDF or TAF
ETV and ADV-resistance	Combination of ETV and TDF, or ETV and TAF

## Conclusion

There are hundreds of millions of people infected with HBV worldwide which causes a huge medical and economic burden. In China, it is expected to eliminate viral hepatitis by 2030. To this end, great efforts have been made by scholars domestically and abroad. In this review, we review the guidelines about who should be treated and try to the most extent to provide some references about how to deal with some patients who fail to meet the treatment standards and the controversial problems during follow-up. There are still many researches need to be done to better address these issues. The controversy over when antiviral therapy should be discontinued in CHB patients is partly due to the lack of effective methods for evaluating cccDNA in the liver cell nucleus (Caviglia et al., 2018; Martinez et al., 2021; Wong et al., 2022). In recent years, growing evidence has identified HBcAg and HBV RNA as favorable biomarkers as an alternative in assessing antiviral efficacy, decision-making on NA withdrawal, and predicting the risk of recurrence (Giersch et al., 2017; Wu et al., 2017; Xie et al., 2021). Huang et al. (2021) reported that the duration for the replacement of the cccDNA pool ranged between several months to 1 year. This finding hinted that short-term cccDNA clearance could be achieved with the cooperation of NAs and cccDNA corepressor. Nevertheless, the sample size of the study was small; thus, some confounding factors, such as serum HBV DNA contamination, were excluded. Additional studies are required to mine the biological characteristics of cccDNA, including synthesis, renewal, and epigenetics, in order to develop a therapy for CHB that can directly target cccDNA.

Previous studies demonstrated that HBV-specific CD4+/CD8+ T cell dysfunction or depletion plays a vital role in persistent infection caused by HBV (Fisicaro et al., 2017; Polaris Observatory Collaborators, 2018; Yong et al., 2018; Pan et al., 2020; Huang et al., 2021; Fang et al., 2022; Li et al., 2022; Zhang et al., 2022). In a recent study by Zhang et al., the single-cell immune sequences of 0.243 million cells from 46 pairs of peripheral blood and liver samples of 23 patients were analyzed, and the dynamic alterations of T cell depletion after HBV infection were profiled (Zhang et al., 2022). In addition to T cell depletion, a decline in the functions and number of DCs and NKs/NKTs augmented the expression of negative regulators of immune checkpoint proteins (such as the PD-1 and cytotoxic T-lymphocyte antigen 4). The innate immunocompromise represented by Toll-like receptors also participated in persistent HBV infection. Thus, breaking immune tolerance and restoring the HBV-specific immune response might be conducive to facilitating HBV control and clearance (Rehermann, 2013). A large number of agents have been developed for different life stages of HBV, such as cccDNA-targeting agents, blockers for HBsAg efflux, Toll-like receptor agonists, immune checkpoint inhibitors, and therapeutic vaccines (Akbar et al., 2012; Park et al., 2016; Rivino et al., 2018; Salimzadeh et al., 2018). Therefore, we believe that drugs that can cure hepatitis B will be developed in the near future, owing to the continuous advancement of technologies.

## Author contributions

TL, JZ, and PX collected the data. LL and YS contributed to figures and tables. MZ and HW edited the manuscript. YW reviewed the manuscript. All authors contributed to the article and approved the submitted version.
